# Long-term effects of combining anaerobic digestate with other organic waste products on soil microbial communities

**DOI:** 10.3389/fmicb.2024.1490034

**Published:** 2025-01-07

**Authors:** Daniela Mora-Salguero, Denis Montenach, Manon Gilles, Vincent Jean-Baptiste, Sophie Sadet-Bourgeteau

**Affiliations:** ^1^Agroécologie, French National Institute for Agriculture, Food, and Environment (INRAE), Institut Agro, Université Bourgogne, Université Bourgogne Franche-Comté, Dijon, France; ^2^French National Institute for Agriculture, Food, and Environment (INRAE), UE 0871 Service d’expérimentation Agronomique et Viticole, Colmar, France; ^3^Gaz Réseau de France (GRDF), Paris, France; ^4^Institut Agro Dijon, France

**Keywords:** anaerobic digestate, soil microbial communities, high-throughput sequencing, organic fertilizer, inorganic fertilizer

## Abstract

**Introduction:**

Agriculture is undergoing an agroecological transition characterized by adopting new practices to reduce chemical fertilizer inputs. In this context, digestates are emerging as sustainable substitutes for mineral fertilizers. However, large-scale application of digestates in agricultural fields requires rigorous studies to evaluate their long-term effects on soil microbial communities, which are crucial for ecosystem functioning and resilience.

**Material and methods:**

This study presents provides a comparative analysis in long-term field conditions of fertilization strategies combining annual applications of raw digestate with biennial applications of different organic waste products (OWPs)—biowaste compost (BIO), farmyard manure (FYM), and urban sewage sludge (SLU)—and compares them to combinations of the same OWPs with mineral fertilizers. The cumulative effects of repeated OWP applications, paired with two nitrogen sources—organic (digestate) and chemical (mineral fertilizer)—were assessed through soil physicochemical and microbial analyses. We hypothesized that the combined effect varied according to the N-supply sources and that this effect also depended on the type of OWP applied. Soil microbial communities were characterized using high-throughput sequencing targeting 16S and 18S ribosomal RNA genes, following DNA extraction from soil samples collected in 2022, six years after the initial digestate application.

**Results:**

The results indicated that combining OWPs rich in stable and recalcitrant organic matter, such as BIO and FYM, with raw digestate, offers an improved fertilization practice. This approach maintains soil organic carbon (SOC) levels, increases soil phosphorus and potassium content, and stimulates microbial communities differently than nitrogen supplied via mineral fertilizers. While microbial biomass showed no significant variation across treatments, microbial diversity indices exhibited differences based on the type of OWP and nitrogen source. The fertilization strategies moderately influenced prokaryotic and fungal community structures, with distinct patterns depending on the OWP and nitrogen source. Notably, fungal communities responded more strongly to treatment variations than prokaryotic communities.

**Discussion:**

This study provides new insights into the cumulative effects of substituting mineral fertilizers with digestates on soil microbial communities and soil physicochemical parameters. The sustainable development of agroecosystems significantly depends on a better understanding of the complex responses of soil microbial communities to different fertilization regimes. Future research should continue to assess the long-term impact of digestate application on soil microbiota in real agronomic field conditions, considering associated agricultural practices.

## 1 Introduction

Agriculture is central to worldwide policy agendas today due to its importance and relevance in satisfying global needs (OECD/FAO, 2023). Over the past decades, to meet growing demands for food, intensive agricultural practices, such as the massive use of chemical inputs and tillage, have significantly affected soil physicochemical properties and soil biodiversity (Christel et al., 2021; Tsiafouli et al., 2015). Agriculture is undergoing an agroecological transition characterized by adopting new practices to reduce chemical fertilizer inputs and encompasses other aspects, such as energy use and nutrient recycling, to ensure food security and mitigate the adverse effects on adjacent ecosystems and climate change (Boeraeve et al., 2020; Porter et al., 2009). As a sustainable agricultural practice, several studies have highlighted the advantages of organic waste products (OWPs) as substitutes for mineral fertilizers (Allam et al., 2022). The OWPs (e.g., farmyard manure, biowaste compost) improve soil fertility and soil organic matter content, and provide bio-stimulants for crops that, coupled with the slower release of nutrients, result in improved nutrient utilization (Francioli et al., 2016; Lori et al., 2017; Ma et al., 2020). Also aligned with an agroecological transition, a sustainable opportunity emerges in the biogas sector (Karimi et al., 2022). The process can use a wide variety of feedstock (crop residues, animal manure, the organic fraction of municipal and industrial solid waste, or wastewater sludge) (IEA, 2020), producing via the anaerobic digestion of the organic matter two high-value byproducts: a renewable energy source (biogas) and an OWP (anaerobic digestate) with potential agronomic properties as a fertilizer and soil amendment (Nkoa, 2014). Indeed, using digestates as fertilizers can sustain yields equivalent to those obtained in mineral-fertilized soils (Barzee et al., 2019; Riva et al., 2016; Walsh et al., 2018; Zicker et al., 2020). However, it is crucial to understand the effects of employing anaerobic digestates as organic fertilizers before promoting their field application, ensuring soil and environmental preservation (Karimi et al., 2022).

The soil is one of the most important reservoirs of biological diversity on our planet (FAO, ITPS, GSBI, SCBD, and EC, 2020). Microorganisms represent a substantial component of this diversity, with estimates ranging from thousands to millions of species *per* gram of soil (Orgiazzi et al., 2016; Torsvik and Øvreås, 2002). These microbial communities (Archaea, Bacteria, Fungi) play vital functions in numerous soil processes and interactions, contributing to several ecosystem services (Delgado-Baquerizo et al., 2020; Pereira et al., 2018). Their roles include functions in biogeochemical cycles, organic matter mineralization, plant growth and productivity, soil structure maintenance, pathogen regulation, and reduction of soil pollutants (Banerjee and van der Heijden, 2023; Guerra et al., 2021; Hartmann and Six, 2023; Maron et al., 2018). Soil microbial communities are considered ideal for monitoring soil quality due to their pivotal role in ecological services, high sensitivity to environmental disturbances, and short generation time (Djemiel et al., 2022; Hermans et al., 2017). Recent advances in molecular biology have significantly enhanced our ability to explore soil microbial diversity, including taxonomic richness and community composition (Schloter et al., 2018). Despite the complexity inherent in multivariate data analysis, the need of advanced bioinformatic tools, and the sophisticated statistical tests performed for interpretation (Fierer et al., 2021), soil microbiome is now commonly used to assess the impact of agricultural practices and land use on soil quality (Christel et al., 2023; Constancias et al., 2015; Dunn et al., 2021; Tardy et al., 2015).

Most studies assessing the impact of anaerobic digestates on soil microbial parameters have utilized laboratory approaches, such as microcosm and mesocosm experiments (Karimi et al., 2022). These methods are advantageous due to their relative ease of implementation and the ability to control sources of variability. However, while these approaches provide evidence of immediate and short-term responses, they often present gaps that are difficult to correlate with field conditions (Karimi et al., 2022). *In-situ* experiments are an integral approach to real agronomic and pedoclimatic conditions, providing a more comprehensive understanding of the short- (≤ 1 year), mid- (1 to 5 years), and long-term (≥ 6 years) effects of anaerobic digestates, aiming to use them as substitutes for mineral fertilizers (Karimi et al., 2022). Several studies in field conditions have demonstrated that the long-term application of mineral or organic fertilizers significantly influences and induces lasting modifications on soil edaphic properties and soil microbial communities (Francioli et al., 2016; Geisseler and Scow, 2014; Kurzemann et al., 2020; Sadet-Bourgeteau et al., 2018). Generally, mineral fertilizers decrease soil organic carbon (SOC) content and tend to acidify the soil, thus affecting soil microorganisms (Bebber and Richards, 2022; Francioli et al., 2016; Geisseler and Scow, 2014). In contrast, the effects of repeated application of OWPs vary depending on the quantity and quality of the applied product (e.g., dose, organic matter content, C/N ratio, pH) (Cui et al., 2023; Francioli et al., 2016; Luo et al., 2018; Sadet-Bourgeteau et al., 2022). Concerning the studies assessing the long-term effects on soil microbial communities in field conditions of combining organic and inorganic fertilizers, the prevalent treatment involves farmyard manure supplemented with mineral fertilizer. These studies typically reveal increases in soil microbial biomass and diversity compared to purely mineral or purely organic fertilization (Cui et al., 2018; Francioli et al., 2016; Kurzemann et al., 2020; Yue et al., 2016; J. Zhao et al., 2014). Moreover, the impact of digestate application is usually assessed by comparing its effects to those of mineral fertilizers or other traditionally used OWPs (e.g., farmyard manure, slurries, and compost) (Karimi et al., 2022). However, the variability in digestate properties, caused by diverse feedstock compositions, production methods, and inherent soil heterogeneity, makes it difficult to establish generalized conclusions about their impact on soil microorganisms (Karimi et al., 2022; van Midden et al., 2023). For instance, multi-year field experiments following applications of liquid or whole digestate have reported no increases in soil microbial biomass (Bhogal et al., 2018; Johansen et al., 2015; Pastorelli et al., 2021; Šimon et al., 2015), nor significant changes in soil bacterial or fungal diversity compared to mineral fertilization (Coelho et al., 2020; Pastorelli et al., 2021; Tang et al., 2021).

The PROspective field experiment was conducted in northeastern France and was set up in 2000. Its primary objective is to systematically evaluate the agronomic value and environmental impact of OWPs in the context of land application policies. The present study aimed to assess the effect of anaerobic digestate combined with different OWPs (digestate acts as an additional N-supply source) on soil microbial communities (Archaea, Bacteria, and Fungi), using molecular DNA-based tools to quantify soil microbial biomass, diversity, and community structure. We hypothesized that (i) soil physicochemical properties and soil microbial parameters (such as molecular microbial biomass, diversity, community structure, and composition) are subject to changes due to the repeated application of anaerobic digestate as an organic fertilizer, and these modifications differ when a traditional inorganic mineral fertilizer is used instead, and (ii) the cumulative long-term effects of repeated applications of distinct OWPs, combined with two different additional sources of N-supply—via an organic source (digestate) or a chemical source (mineral fertilizer)—vary significantly, but these differences depend on the type of OWP applied. This study allowed us to explore and analyze in field conditions, the effects on soil microbial communities of an enhanced fertilization practice. This practice is based on combining the repeated annual application of anaerobic digestate with biennial applications of other traditional OWPs (such as farmyard manure, biowaste compost, and sewage sludge).

## 2 Materials and methods

### 2.1 Experimental site, soil sampling strategy, and soil chemical analysis

The study was carried out at the PROspective experimental site (Colmar, France; 48°03′33N, 7°19′42″E) of the SOERE-PRO^1^ network, designed for long-term studies of the evolution of agrosystems after repeated applications of OWPs. The climate is semi-continental, with a mean annual precipitation of 569 mm received mostly between May and October and an 11.3°C mean annual temperature. According to the World Reference Base for Soil Resources, the soil is classified as Calcosol (125.5 g kg^−1^ calcium carbonate in the plowed layer) (Anjos et al., 2015). The experimental site was set up in 2000. The topsoil horizon had the following physicochemical properties at the beginning of the trial: 21% clay; 70% silt; 9% sand; pH 8.3; total N 1.4 g kg^−1^; soil organic carbon (SOC) 14.3 g kg^−1^; and Olse-P (NaHCO_3_-extractable P) 31 mg kg^−1^. Since 2001, the crop succession was maize (*Zea mays*)-winter wheat (*Triticum aestivum*)-sugarbeet (*Beta vulgaris*)-spring barley (*Hordeum vulgare*), except for 2003, when maize was sown instead of sugarbeet. All aboveground crop residues were returned to the soil. Since 2014, mustard (*Sinapis alba*) has been used as a cover crop before maize and sugarbeet.

The field experiment was conducted in 32 plots, a complete randomized block design of 8 treatments and 4 replicates, comparing four different OWPs. Each 9 m x 10 m plot was separated by 6 m wide cultivated bands and the blocks by 10 m wide cultivated strips to avoid cross-contamination. The treatments were arranged in two sub-trials based on a nitrogen supply strategy: (i) OWP without mineral N supply (16 plots) and (ii) OWP with mineral N supply (16 plots) (Supplementary Figure 1). The OWPs were as follows: urban sewage sludge (SLU) derived from the SITEUCE wastewater treatment plant, biowaste compost (BIO) made from the home-sorted organic fraction of municipal solid waste co-composted with green waste, farmyard manure (FYM) made from a mix of dairy cow feces and urine with cereal straw, and raw digestate (DIG) derived from anaerobic digestion of 60% biowastes (restaurants and agri-food industries), 20% livestock effluents, and 20% plant matter.

From 2001 to 2013, every 2 years in early spring, the same amount of SLU, BIO, and FYM was applied (approximately 170 kg N ha^−1^; the maximal annual amount allowed by EU legislation) in both sub-trials. Since 2014, the fertilization strategy was modified to achieve similar yields in both sub-trials. For this purpose, DIG has been applied in all plots at the sub-trial without mineral N supply as a complement when additional available N is required to reach equivalent yields to those plots at the sub-trial with mineral N supply. The control plots in the sub-trial without mineral N supply did not receive any OWP input from 2001 to 2014. Since 2015, these control plots have received an annual DIG input. Since 2016, the amounts of SLU, BIO, and FYM were adjusted at each application based on their chemical characteristics (N content and potentially available N) and the requirements of the spring crop. Since 2001 in the sub-trial with mineral N supply, the control plots (MIN) followed yearly mineral fertilization (ammonium nitrate); for the other plots of this sub-trial, the mineral fertilizer was applied once or twice yearly depending on the treatment, and the quantity was adjusted for each treatment as a function of the amount of mineral N in the soil (Chen et al., 2022). The latest applications before soil sampling (soil sampling performed in April 2022) were in April 2021 for DIG, May 2021 for MIN, and in both sub-trials in January 2022 for SLU, BIO, and FYM. As it stands, the total set of treatments by sub-trial was (i) OWPs without mineral N supply: BIO-DIG, FYM-DIG, SLU-DIG, and DIG; and (ii) OWPs with mineral N supply: BIO-MIN, FYM-MIN, SLU-MIN, and MIN. The mean OWP characteristics applied from 2001 to 2022 are given in Table 1. The index of residual organic carbon (I_ROC_) represents the proportion of the OWP contributing to soil organic C storage (Lashermes et al., 2009). The detailed amounts of organic carbon and fertilizing elements (NPK) applied over the 2001–2022 period at each plot are presented in Supplementary Tables 1–3 and Supplementary Figure 2.

**Table 1:** Mean characteristics of the organic waste products (OWPs) applied at the PROspective (experimental field site in Colmar, France).

OWP	Applied quantity	Dry matter (DM)	Organic carbon	I_ROC_	Total N	Mineral N	C:N	P_2_O_5_ (total HF)	K_2_O (total HF)	pH
	t DM ha^−1^	%	kg ha^−1^	% Organic C	kg ha^−1^			kg ha^−1^		
BIO	9.7a (4.3)	57.8a (18.6)	2554b (1375)	68.8a (9.7)	200a (99.7)	14.9c (15.1)	12.7b (1.8)	111b (66)	245b (105)	8.2b (0.5)
FYM	8.6a (3.8)	24.2b (7.4)	3452a (1553)	55.8b (7.6)	198a (76.3)	28.3c (27.9)	17.7a (4.3)	117b (47.9)	383a (160)	9.5a (0.3)
SLU	2.6b (0.6)	18.2b (1.7)	972c (179)	48.6c (8.9)	156a (30.7)	51.7b (26.4)	6.3c (0.4)	177a (49.4)	20.6d (5.8)	7.1c (0.6)
DIG	1.8b (0.5)	6.2c (1.4)	607c (218)	50.8bc (8.9)	183a (86.4)	135.5a (40.2)	3.7d (0.5)	59.4c (16.8)	109c (54)	8.4b (0.4)

OWPs: urban sewage sludge (SLU) derived from the SITEUCE wastewater treatment plant, biowaste compost (BIO) made from the home-sorted organic fraction of municipal solid waste co-composted with green waste, farmyard manure (FYM) made from a mix of dairy cow feces and urine with cereal straw, and raw digestate (DIG) derived from anaerobic digestion of 60% biowastes (restaurants and agri-food industries), 20% livestock effluents, and 20% plant matter.

The mineral N represents the sum of N in forms NH_4_^+^ and NO_3_^−^.

Values are means, and letters denote the significant effect between treatments (*p* < 0.05) based on Tukey’s HSD test *p*-value adjusted by the BH method. The standard error of the means is indicated in parentheses.

As previously mentioned, soil samples were collected in April 2022. Each sample comprises 8 soil cores, extracted at random locations in each plot from the 0–20 cm horizon, then mixed and homogenized by 4 mm mesh sieving to remove above-ground plant debris, roots, and stones. The sieved soil was lyophilized and stored at −40°C prior to DNA extraction and molecular analysis. A portion of each soil was air-dried for physicochemical analysis: particle size distribution, pH, soil organic carbon, soil total nitrogen, total phosphorus, and Cation Exchange Capacity (CEC). These analyses were performed at the INRAE Soil Laboratory Analysis.^2^ The measured soil physicochemical data is given in Table 2.

**Table 2:** Means of soil physicochemical properties (0-20 cm horizon) at the PROspective (experimental field site in Colmar, France) according to different fertilization treatments.

Treatment	Organic carbon	Total *N*	P_2_O_5_ (Olsen)	K_2_O	C:N	pH	CEC
	g kg^−1^						cmol + kg^−1^
BIO-DIG	16.2a (1.03)	1.57a (0.1)	0.07c* (0.01)	0.4b* (0.06)	10.4bc (0.20)	8.33ab (0.04)	16.0a (0.5)
BIO-MIN	15.4a (0.53)	1.47b (0.06)	0.05d* (0.004)	0.3c* (0.04)	10.5b (0.15)	8.29bc (0.02)	16.3a (0.5)
FYM-DIG	15.4a* (0.94)	1.44b (0.07)	0.09ab* (0.006)	0.6a* (0.05)	10.7ab (0.47)	8.34ab (0.02)	16.0a* (0.3)
FYM-MIN	14.3b* (0.30)	1.38bd (0.05)	0.07c* (0.004)	0.4b* (0.03)	10.4bc (0.27)	8.31b (0.02)	16.6a* (0.4)
SLU-DIG	14.1b (0.56)	1.31 cd (0.04)	0.09a* (0.006)	0.2d (0.02)	10.7ab* (0.18)	8.33ab* (0.02)	16.1a* (0.05)
SLU-MIN	13.4bc (0.07)	1.33 cd (0.04)	0.08b* (0.002)	0.2 cd (0.02)	10.1c* (0.34)	8.25c* (0.03)	16.4a* (0.2)
DIG	13.8bc* (0.43)	1.26c (0.04)	0.05d (0.003)	0.2 cd (0.02)	11.0a* (0.15)	8.37a (0.03)	15.9a* (0.2)
MIN	12.9c* (0.46)	1.25c (0.03)	0.04d (0.007)	0.2 cd (0.01)	10.3bc* (0.16)	8.35ab (0.03)	16.4a* (0.2)

Treatments: urban sewage sludge (SLU), biowaste compost (BIO), farmyard manure (FYM), combined with two different additional sources of N-supply via raw digestate (DIG) or mineral fertilizer (MIN).

Values are means, and letters denote the significant effect between treatments (*p* < 0.05) based on Tukey’s HSD test *p*-value adjusted by the BH method. (*) denote the significance effect (*p* < 0.05) within a pair of counterpart treatments. The standard error of the means is indicated in parentheses.

### 2.2 DNA extraction and purification

DNA extraction from 1 g of soil (dry weight) was carried out using a standardized methodology established by the GenoSol platform (INRAE, Dijon, France)^3^ (Terrat et al., 2012). This protocol comprises three key steps: (i) physical and chemical lysis of microbial cells, (ii) deproteinization, and (iii) precipitation and washing of nucleic acids with alcohol. The concentrations of DNA in the crude extracts were assessed using electrophoresis in a 1% agarose gel stained with ethidium bromide, employing calf thymus DNA as the standard calibration curve. The quantified crude DNA was employed as a proxy for estimating soil molecular microbial biomass (Dequiedt et al., 2011). To mitigate residual impurities, notably humic substances, 100 mL of crude DNA was subjected to purification using the Nucleospin® Soil kit (Macherey-Nagel GmbH & Co. KG, Düren, Germany). Finally, the concentrations of purified DNA were determined using the Quantifluor staining kit (Promega, Madison, Wisconsin, USA), following to the manufacturer’s instructions.

### 2.3 High throughput sequencing of 16S and 18S rRNA gene sequences

The diversity of prokaryotic organisms (bacteria and archaea) within each DNA sample was assessed *via* metabarcoding of the 16S rRNA gene, following the methodology described by Terrat et al. (2015). A 440-based fragment targeting the V3 to V4 regions was amplified using the primer pair F479 (5′CAG CMG CYG CNG TAA NAC3′) and R888 (5′CCG YCA ATT CMT TTR AGT3′). For the evaluation of fungal diversity in each DNA sample, the metabarcoding of the 18S rRNA gene was employed, as described by (Chemidlin Prévost-Bouré et al., 2011), targeting a 350-base fragment from V7 to V8 regions, amplified using primers FR1 (5′ANC CAT TCA ATC GGT ANT3′) and FF390 (5′CGA TAA CGA ACG AGA CCT3′). PCR amplifications for each sample were conducted with 5 ng of DNA in a total reaction volume of 25 μL. The thermal profile for prokaryotic PCR encompassed an initial denaturation at 94°C for 2 min, followed by 35 cycles of denaturation at 94°C for 30 s, annealing at 52°C for 30 s, and extension at 72°C for 1 min, and a final extension at 72°C for 7 min. The thermal profile for fungal PCR comprised an initial denaturation at 94°C for 3 min, followed by 35 cycles of denaturation at 94°C for 30 s, annealing at 52°C for 1 min, and extension at 72°C for 1 min, and a final extension at 72°C for 5 min. Post-PCR, all products were purified using the ProNex® Size-Selective Purification System (Promega, Madison, Wisconsin, USA) and quantified with the Quantifluor staining kit (Promega, Madison, Wisconsin, USA). Subsequently, a second PCR was performed on the purified products (7.5 ng of DNA for bacteria and archaea and 5 ng of DNA for fungi, in a total reaction mix volume of 25 μL), incorporating 10-base-pair multiplex identifiers (MID) at the 5′ and 3′ ends of the primers to sample identification. The thermal profile for the second PCR was similar to the first, with adjustments made for bacterial/archaeal libraries (7 cycles) and fungal libraries (7 cycles and a denaturation step of 94°C for 1 min). Following the second PCR, products were purified using the MinElute PCR purification kit (Qiagen NV) and quantified with the Quantifluor staining kit (Promega, Madison, Wisconsin, USA). Samples were then equimolarly pooled and further purified using the ProNex® Size-Selective Purification System (Promega, Madison, Wisconsin, USA) to remove excess nucleotides, salts, and enzymes. Finally, sequencing was performed using the NovaSeq Illumina instrument (Illumina Inc., San Diego, California, USA), generating 250-bp paired-end reads.

### 2.4 Bioinformatic analysis of 16S and 18S rRNA gene sequences

Bioinformatic analyses were conducted using the BIOCOM-PIPE pipeline (Djemiel et al., 2020). All raw reads 16S and 18S were initially sorted based on their MID sequences. Data preprocessing involved the initial trimming of raw reads *via* PRINSEQ, followed by merging paired-end reads using FLASH. Low-quality reads were discarded based on predefined criteria, including minimum length, number of ambiguities (Ns), and primer sequences. Subsequently, reads were dereplicated to optimize computational efficiency in further pipeline steps, specifically for clustering identical sequences. Dereplicated reads were aligned using the Infernal tool (Nawrocki and Eddy, 2013), followed by clustering into operational taxonomic units (OTUs) using a similarity threshold of 95%; this threshold is adapted for the targeted amplicon and previously justified (Djemiel et al., 2020; Terrat et al., 2020). A chimera filtering step was implemented based on the quality of taxonomic alignments (Djemiel et al., 2020). Subsequently, high-quality reads were standardized by randomly selecting 10,000 reads to ensure dataset comparability and mitigate biased community comparisons. The retained reads were employed for (i) post-clustering step using ReClustOR tool (Terrat et al., 2020) to enhance OTU consistency based on a reference OTU database derived from the RMQS project (French Soil Quality Monitoring Network) (Terrat et al., 2017); (ii) taxonomy-independent analyses to compute diversity indices (e.g., richness, Shannon, Inverse Simpson) using the OTU dataset; and (iii) taxonomy-based analysis by similarity approaches against curated reference databases from SILVA r132 (Quast et al., 2013). The raw datasets associated with this study are accessible within the EBI database system under project accession number PRJEB79399.

### 2.5 Statistical analyses

Statistical analyses were conducted with the free statistical software R (R version 4.2.2, 2022-10-31) using R Studio (RStudio, Version 2022.12.0 + 353, Posit Software, PBC formerly RStudio, Boston, Massachusetts, USA). OWPs characteristics, soil physicochemical parameters, and soil molecular microbial biomass were subjected to analysis of variance (ANOVA), followed by a Tukey’s HSD *post hoc* test with the Benjamini & Hochberg (BH) correction method to adjust *p*-values. Alpha diversity indices were subjected to Kruskal-Wallis test, followed by a Dunn post hoc test with the Benjamini & Hochberg (BH) correction method to adjust *p*-values. The significance threshold was set at *p*-0.05. Alpha diversity was assessed using Hill numbers generated by the *vegan* package (Oksanen et al., 2022). Hill numbers facilitate the linear interpretation of alpha diversity by systematically considering the abundances of rare and dominant OTUs using the scaling parameter “q” (order of diversity) (Alberdi and Gilbert, 2019). Precisely, *q* = 0 corresponds to species richness, *q* = 1 represents the exponential of Shannon entropy, where OTUs are weighted by their frequency without disproportionately favoring rare or abundant ones, and *q* = 2 represents the inverse of Simpson index, where the abundant OTUs are overweighed (Alberdi and Gilbert, 2019). In this study, the similarities in the composition of soil prokaryotic and fungal communities among different fertilization treatments were evaluated based on the robust Aitchison dissimilarity distance (Martino et al., 2019), using the analysis of similarities (ANOSIM) and the analysis of multivariate homogeneity of group dispersions (beta-dispersion), employing 999 permutations and a significance threshold of *p* < 0.05. To visualize the distribution patterns of microbial communities for each fertilization treatment based on the robust Aitchison dissimilarity distance (Martino et al., 2019), the non-metric multidimensional scaling (NMDS) approach was employed using the *metaMDS* function from the *vegan* package (Oksanen et al., 2022). To better understand the sources of variations of the composition of microbial communities, phylum, soil physico-chemical properties and fertilization practices data were fitted in the NMDS space using the *envfit* function (*vegan* package; 1,000 permutations) (Oksanen et al., 2022). Only the variables with *p* < 0.001 were retained and plotted in the NMDS space. A non-parametric permutational multivariate analysis of variance (PERMANOVA) based on the robust Aitchison dissimilarity distance (Martino et al., 2019) was used to assess the significance of differences in the structure of soil microbial communities between fertilization treatments. Relative effects of each treatment were tested using the *adonis2* function from the *vegan* package (Oksanen et al., 2022), with 999 permutations and a significance threshold of *p* < 0.05. Finally, the abundance of soil prokaryotic and fungal *phyla* was subjected to a differential abundance analysis comparing counterpart treatments using the DESeq2 method (Love et al., 2014).

## 3 Results

### 3.1 OWPs and soil physicochemical properties

The OWPs highlighted different chemical properties (*p* < 0.05, Table 1 and Supplementary Figure 3). All the OWPs had pH values greater than 7, and FYM showed the highest pH (9.5), whereas SLU presented the lowest pH (7.1). BIO and FYM were characterized by higher organic carbon, I_ROC_, C/N ratio, and K_2_O contents compared to SLU and DIG (*p* < 0.05, Table 1). Moreover, although FYM presented a significantly higher organic carbon content (*p* < 0.05, Table 1), BIO showed a higher potential to contribute to soil C storage (I_ROC_; *p* < 0.05, Table 1). The digestate applied (DIG) was a liquid product characterized by the highest mineral N content (sum of N in forms NH_4_^+^ and NO_3_^−^) and lowest values in dry matter content, C/N ratio, and P_2_O_5_ content among the OWPs (*p* < 0.05, Table 1).

The soil physicochemical properties were strongly influenced by the long-term and repeated application of diverse OWPs (BIO, FYM, and SLU) combined with two different additional sources of N-supply (DIG or MIN) (Table 2). Plots amended with biowaste compost (BIO-DIG and BIO-MIN) arbored higher values of soil organic carbon content (SOC) (approximately 15% more) compared to plots fertilized only with DIG or MIN (*p* < 0.05, Table 2). The effect of the additional source of N-supply *via* DIG or MIN on the SOC content was only observed in plots receiving farmyard manure (FYM-DIG and FYM-MIN), where the repeated application of farmyard manure combined with digestate inputs (FYM-DIG) highlighted a higher SOC content (approximately 7% more) compared to its respective counterpart treatment (FYM-MIN) (*p* < 0.05, Table 2). Overall, considering SOC across all plots, the descending order by couples of treatments was BIO > FYM > SLU > Control (*p* < 0.05, Table 2).

Similarly, soil total N content followed the same trend (BIO > FYM > SLU > Control) (*p* < 0.05, Table 2). However, no significant differences in total N content were observed between control plots (receiving only DIG or MIN) (*p* > 0.05, Table 2). The additional nitrogen source *via* DIG or MIN had a significant effect on soil total N content only in plots receiving biowaste compost (BIO-DIG and BIO-MIN), where the repeated application of biowaste composts combined with digestate inputs (BIO-DIG) exhibited approximately 7% higher soil total N content compared to BIO-MIN treated plots (*p* < 0.05, Table 2). The soil C/N ratios ranged from 10.1 to 11; plots amended only with DIG recorded a significantly higher C/N ratio (approximately 7% more) compared to plots receiving only mineral inputs (MIN) (*p* < 0.05, Table 2). The effect of additional source of N-supply *via* DIG or MIN on the soil C/N ratio was only observed in urban sewage sludge treated plots (SLU-DIG and SLU-MIN), where the repeated application of SLU combined with digestate inputs (SLU-DIG) reflected a significant higher C/N ratio (approximately 6% more) compared to its respective counterpart treatment (SLU-MIN) (*p* < 0.05, Table 2). Regarding soil pH, values ranged between 8.25 and 8.37; no significant differences were observed between the control plots (those receiving only DIG or only MIN) (*p* > 0.05, Table 2). The effect of an additional source of N-supply was observed only in SLU-treated plots, where the repeated application of SLU combined with digestate inputs (SLU-DIG) exhibited a slightly higher but significant pH value compared to SLU-MIN treated plots (*p* < 0.05, Table 2).

### 3.2 Soil molecular microbial biomass and diversity indices

**Figure 1:**
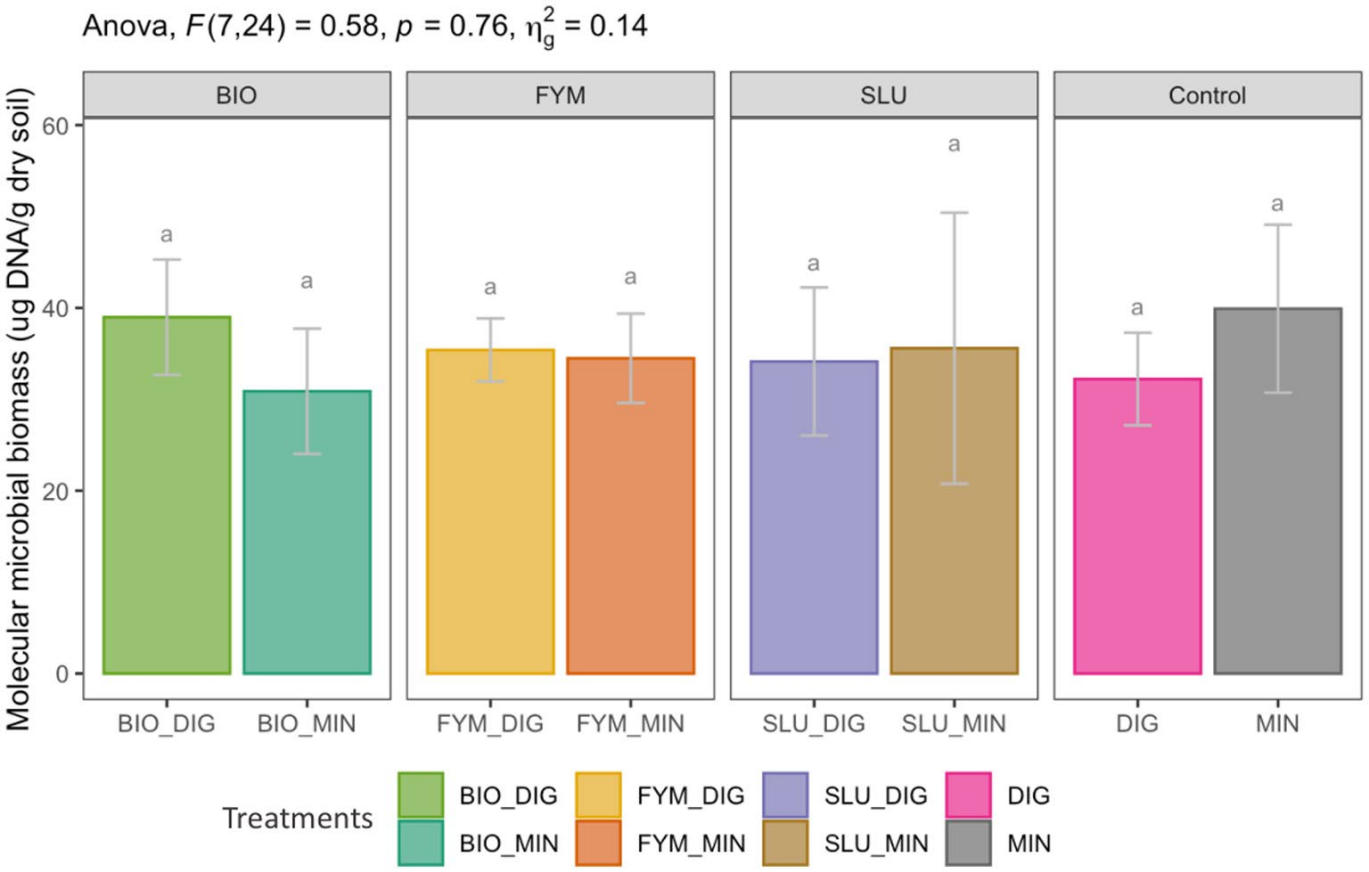
Effect of different fertilization strategies on soil molecular microbial biomass at the PROspective (experimental field site in Colmar, France). Treatments: urban sewage sludge (SLU), biowaste compost (BIO), farmyard manure (FYM), combined with two different additional sources of N-supply via raw digestate (DIG) or mineral fertilizer (MIN). Lower-case letters: effect between treatments, different letters indicate significant differences (*p* < 0.05) based on the Tukey’s HSD test; *p*-value adjusted by the BH method.

Different fertilization strategies resulted in equivalent soil molecular microbial biomass (*p* > 0.05, Figure 1). The field application of biowaste compost, farmyard manure, or urban sewage, supplemented with additional N inputs via an organic source (digestate) or a chemical source (mineral fertilizer), induced a similar effect on the soil molecular microbial biomass (*p* > 0.05, Figure 1).

**Figure 2:**
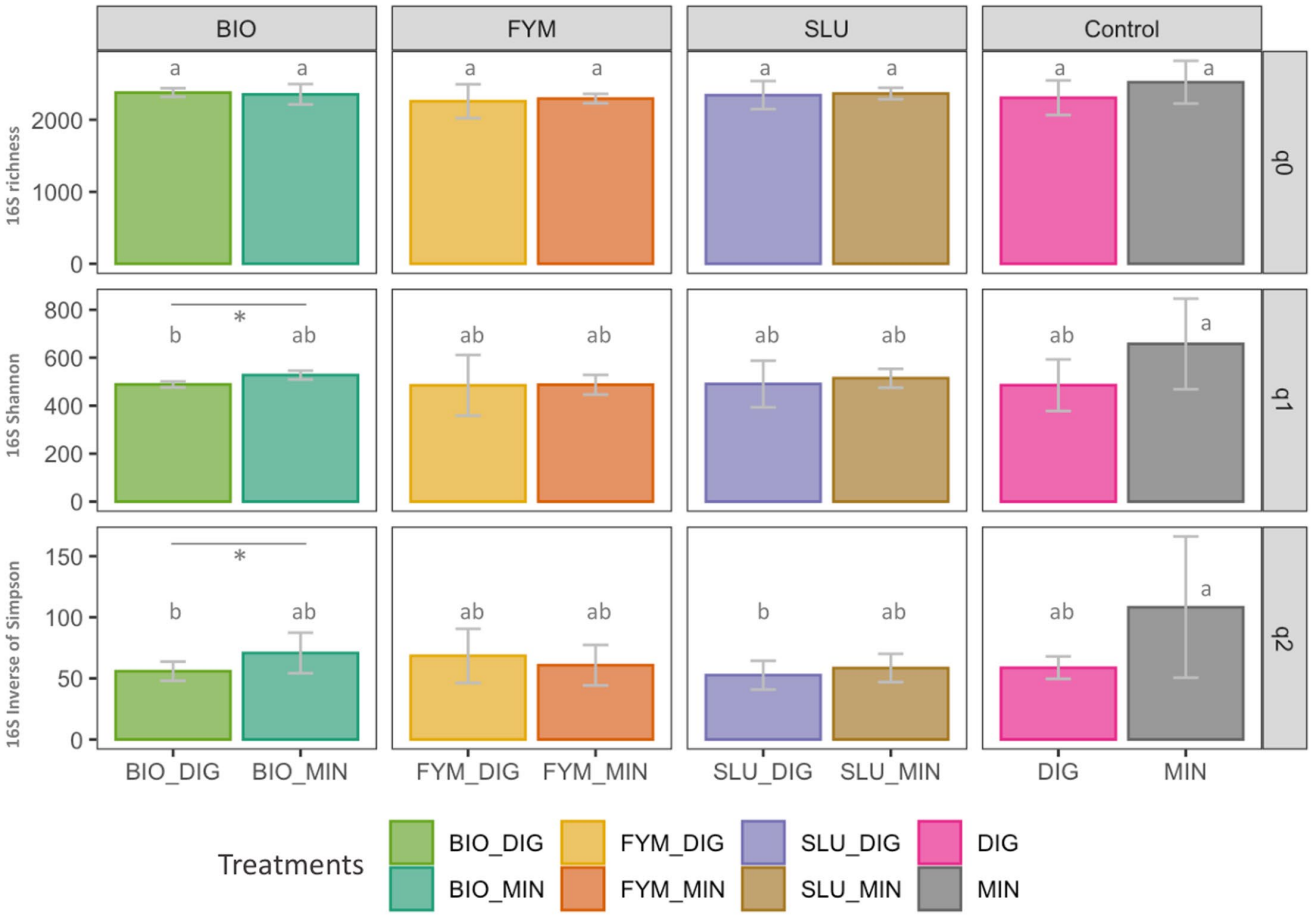
Prokaryotic community diversity in soil based on Hill numbers at the PROspective (experimental field site in Colmar, France). Hill numbers facilitate the linear interpretation of alpha diversity by progressively considering the abundances of rare and dominant OTUs using the scaling “q” parameter. **(A)** species richness (Hill number *q* = 0; q0 represent the number of OTUs), **(B)** Shannon index (Hill number *q* = 1; q1 represents the exponential of Shannon entropy), and **(C)** inverse of Simpson index (Hill number *q* = 2). Treatments: urban sewage sludge (SLU), biowaste compost (BIO), farmyard manure (FYM), combined with two different additional sources of N-supply *via* raw digestate (DIG) or mineral fertilizer (MIN). Lower-case letters: effect between treatments, different letters indicate significant differences (*p* < 0.05) based on the Kruskal-Wallis test; *p*-value adjusted by the BH method. *Significant effect (*p* < 0.05) within a pair of counterpart treatments.

**Figure 3:**
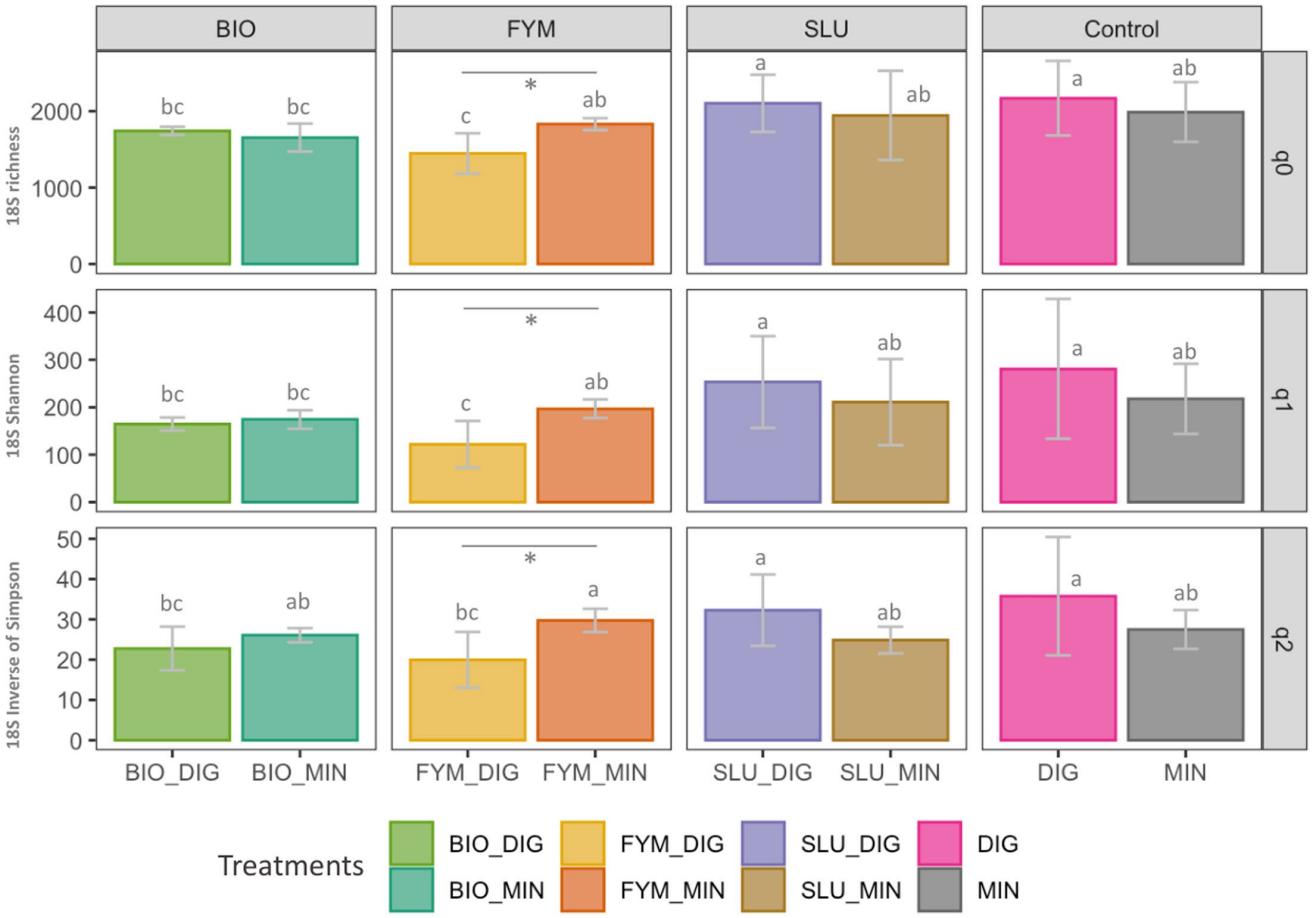
Fungal community diversity in soil based on Hill numbers at the PROspective (experimental field site in Colmar, France). Hill numbers facilitate the linear interpretation of alpha diversity by progressively considering the abundances of rare and dominant OTUs using the scaling “q” parameter. **(A)** species richness (Hill number *q* = 0; q0 represent the number of OTUs), **(B)** Shannon index (Hill number *q* = 1; q1 represents the exponential of Shannon entropy), and **(C)** inverse of Simpson index (Hill number *q* = 2). Treatments: urban sewage sludge (SLU), biowaste compost (BIO), and farmyard manure (FYM), combined with two different additional sources of N-supply *via* raw digestate (DIG) or mineral fertilizer (MIN). Lower-case letters: effect between treatments, different letters indicate significant differences (*p* < 0.05) based on the Kruskal-Wallis test; *p*-value adjusted by the BH method. *Significant effect (*p* < 0.05) within a pair of counterpart treatments.

No differences were observed in prokaryotic or fungal diversity indices between plots receiving only digestate inputs (DIG) or only mineral fertilizer inputs (MIN). The long-term effect of repeated applications of distinct OWPs (BIO, FYM, and SLU) combined with two different additional sources of N-supply, i.e., DIG or MIN, exhibited some differences in prokaryotic and fungal diversity indices depending on the type of the OWP applied. In the prokaryotic community, a significant response within pairs of counterpart treatments was observed only in BIO couple plots, where BIO-DIG plots showed lower OTUs frequency (*q* = 1) and lower effective number of dominant OTUs (*q* = 2) compared to BIO-MIN plots (*p* < 0.05, Figure 2). Regarding the fungal community, significant differences within pairs of counterpart treatments were observed only in FYM couple plots, where FYM-DIG induced lower values over all indices compared to FYM-MIN plots (*p* < 0.05, Figure 3).

### 3.3 Soil microbial community structure

The analysis of similarities (ANOSIM) and multivariate homogeneity of group dispersions (beta-dispersion) revealed distinct patterns in the prokaryotic and fungal communities in response to the different fertilization treatments. For the prokaryotic community, treatments resulted in moderate separation (*R* = 0.15, *p* = 0.003; Table 3), with significant differences in beta-dispersion (*p* = 0.002; Table 3), indicating variability within treatments. However, the counterpart treatments showed a weak separation (*R* = 0.07, *p* = 0.03; Table 3) and no significant difference in beta-dispersion (*p* = 0.8), suggesting limited differentiation among counterpart treatments. The sub-trial showed no significant separation (*R* = 0.03, *p* = 0.13; Table 3) or differences in beta-dispersion (*p* = 0.8; Table 3), indicating a minimal effect of this factor on prokaryotic community structure.

**Table 3:** Analysis of similarities (ANOSIM) and analysis of multivariate homogeneity of group dispersions (beta-dispersion) depicting the differences in the prokaryotic and fungal community structures based on the Robust-Aitchison distance matrices at the PROspective (experimental field site in Colmar, France), according to the treatment, pairs of counterpart treatments, and sub-trial (999 permutations, significance threshold *p* < 0.05).

	Prokaryotic community			Fungal community		
	ANOSIM		Beta-dispersion	ANOSIM		Beta-dispersion
	R statistic	*p*-value	*p*-value	R statistic	*p*-value	*p*-value
Treatment	0.15	0.003	0.02	0.32	0.0009	0.38
Counterpart treatments	0.07	0.03	0.8	0.19	0.0009	0.38
Sub-trial	0.03	0.13	0.8	0.15	0.004	0.46

Treatments: urban sewage sludge (SLU), biowaste compost (BIO), and farmyard manure (FYM), combined with two different additional sources of N-supply via raw digestate (DIG) or mineral fertilizer (MIN). By sub-trial: (i) OWPs without mineral N supply: BIO-DIG, FYM-DIG, SLU-DIG, and DIG; and (ii) OWPs with mineral N supply: BIO-MIN, FYM-MIN, SLU-MIN, and MIN.

R statistic: degree de separation between test groups ranging from-1 to 1; *R* = 0, not different; *R* = 1, completely different. Significance values were based on 999 permutations and threshold *p* < 0.05.

In contrast, the fungal community showed a more pronounced response to the treatments, with a moderate separation (*R* = 0.32, *p* = 0.0009; Table 3) and no significant difference in beta-dispersion (*p* = 0.38; Table 3), suggesting that the treatments affect the overall community structure, but do not significantly alter the internal variability. The counterpart treatments also showed a moderate separation (*R* = 0.19, *p* = 0.0009; Table 3) with no significant differences in beta-dispersion (*p* = 0.38; Table 3). Similarly, the sub-trial showed moderate differentiation (*R* = 0.15, *p* = 0.004; Table 3) without significant differences in beta-dispersion (*p* = 0.46; Table 3). These findings indicate that the fungal community structure is more responsive to treatment types than the prokaryotic community structure, and their internal variability within each treatment remains similar.

**Figure 4:**
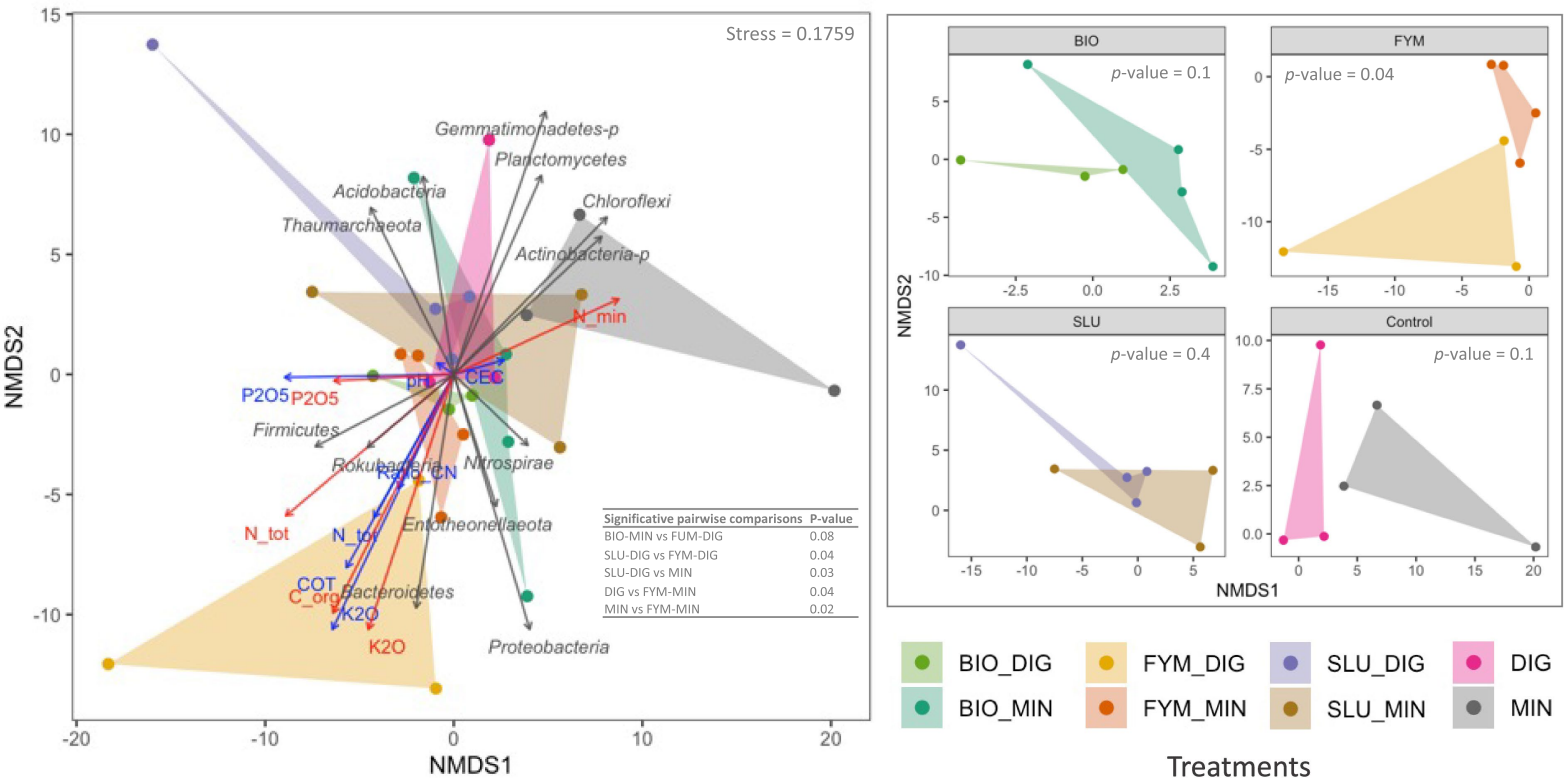
Non-metric multidimensional scaling (NMDS) ordination derived from robust Aitchison dissimilarity distances for the soil prokaryotic community at the PROspective (experimental field site in Colmar, France). Treatments: urban sewage sludge (SLU), biowaste compost (BIO), and farmyard manure (FYM), combined with two different additional sources of N-supply *via* raw digestate (DIG) or mineral fertilizer (MIN). Permutational multivariate analysis of variance (PERMANOVA) depicting the significative differences in prokaryotic structure according to the fertilization treatment (999 permutations, significance threshold *p* < 0.05). **(A)** prokaryotic community structure, gray arrows indicate the major prokaryotic phyla, blue arrows denote the soil physicochemical properties, red arrows represent the cumulative total inputs of organic carbon, nitrogen (total and mineral), phosphorus and potassium, and **(B)** prokaryotic community structure by pairs of counterpart treatments.

**Figure 5:**
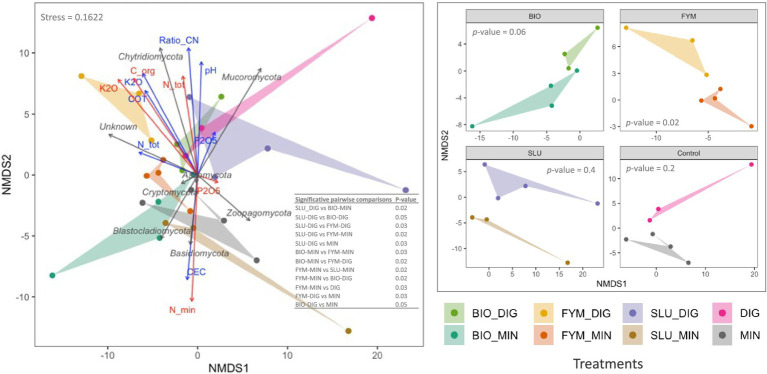
Non-metric multidimensional scaling (NMDS) ordination derived from robust Aitchison dissimilarity distances for the soil fungal community at the PROspective (experimental field site in Colmar, France). Treatments: urban sewage sludge (SLU), biowaste compost (BIO), and farmyard manure (FYM), combined with two different additional sources of N-supply *via* raw digestate (DIG) or mineral fertilizer (MIN). Permutational multivariate analysis of variance (PERMANOVA) depicting the significative differences in prokaryotic structure according to the fertilization treatment (999 permutations, significance threshold *p* < 0.05). **(A)** fungal community structure, gray arrows indicate the major fungal phyla, blue arrows denote the soil physicochemical properties, red arrows represent the cumulative total inputs of organic carbon, nitrogen (total and mineral), phosphorus and potassium, and **(B)** fungal community structure by pairs of counterpart treatments.

NMDS ordination confirmed these findings, clearly highlighting distinct clustering by treatment and reflecting that the quality of the applied products and the soil physicochemical properties shaped the structure of the microbial communities (Figures 4A, 5A). PERMANOVA analysis indicated that the fertilization treatment significantly affected the structure of both prokaryotic and fungal communities (Figures 4A, 5A and Supplementary Tables 4, 5; *p* < 0.05). Pairwise comparisons of counterpart treatments revealed significant differences in prokaryotic community structure only between FYM-MIN and FYM-DIG treated plots (*p* < 0.05; Figure 4B). In contrast, significant differences were observed for the fungal community structure between BIO-MIN and BIO-DIG treated plots, as well as between FYM-MIN and FYM-DIG treated plots (*p* < 0.05; Figure 5B).

**Figure 6:**
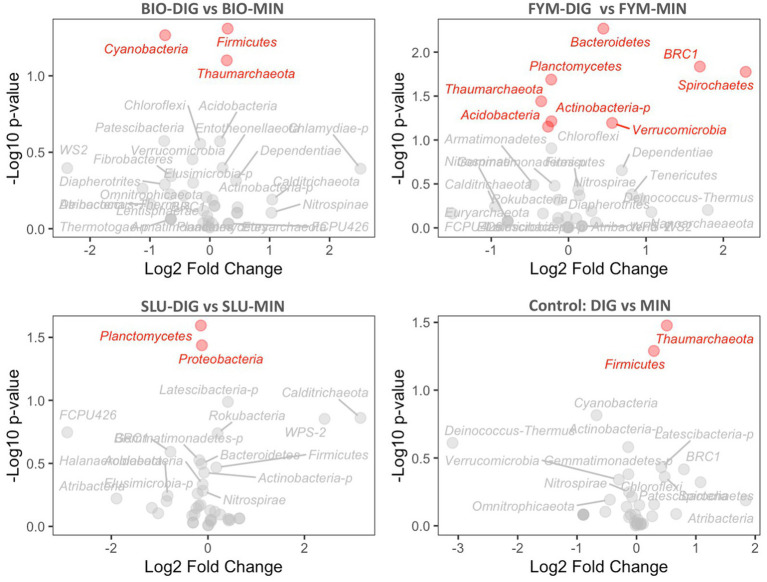
Prokaryotic phyla differential abundance analysis comparing counterpart treatments using the DESeq2 method, showing the relationship between the differential expression value (log2FoldChange) and the statistical significance (*p*-value). **(A)** BIO-DIG vs. BIO-MIN, **(B)** FYM-DIG vs. FYM-MIN, **(C)** SLU-DIG vs. SLU-MIN, **(D)** DIG vs. MIN. A log2FoldChange positive value indicates higher abundance compared to a reference condition (the reference condition was the treatment in the sub-trial with mineral N-supply: BIO-MIN, FYM-MIN, SLU-MIN, and MIN). Red phyla indicate the significant effect within a pair of counterpart treatments.

**Figure 7:**
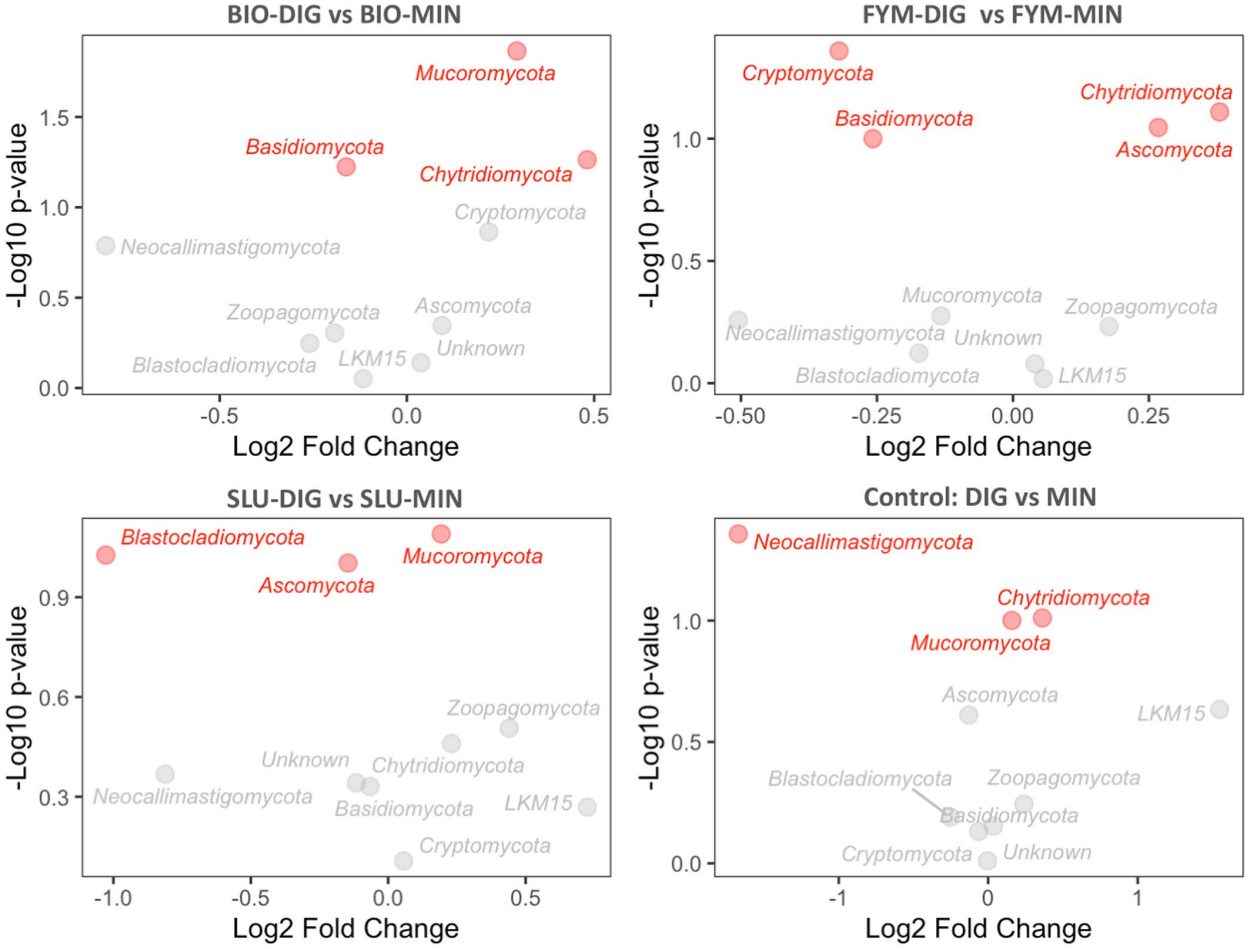
Fungal phyla differential abundance analysis comparing counterpart treatments using the DESeq2 method, showing the relationship between the differential expression value (log2FoldChange) and the statistical significance (p-value). **(A)** BIO-DIG vs. BIO-MIN, **(B)** FYM-DIG vs. FYM-MIN, **(C)** SLU-DIG vs. SLU-MIN, **(D)** DIG vs. MIN. A log2FoldChange positive value indicates higher abundance compared to a reference condition (the reference condition was the treatment in the sub-trial with mineral N-supply: BIO-MIN, FYM-MIN, SLU-MIN, and MIN). Red phyla indicate the significant effect within a pair of counterpart treatments.

The differential abundance analysis conducted using the DESeq2 method provided insights into the changes in the abundance of various microbial phyla in response to the fertilization treatments applied. The long-term effects of repeated applications of different OWPs (BIO, FYM, and SLU) combined with two different additional N-supply sources *via* DIG or MIN exhibited some differences at the phylum level in prokaryotic and fungal communities (Figures 6, 7, Supplementary Figures 4, 5). For prokaryotic phyla, *Thaumarchaeota* and *Firmicutes* exhibited relatively higher base mean counts, indicating their higher abundance when comparing DIG *versus* MIN, and BIO-DIG *versus* BIO-MIN treated plots (Figures 6A,D and Supplementary Figure 4). *Planctomycetes* and *Proteobacteria* only exhibited slight but significant changes in SLU-treated plots (SLU-DIG vs. SLU-MIN) (Figure 6C). When comparing FYM-DIG treated plots *versus* FYM-MIN treated plots, significantly higher levels were observed in *Bacteroidetes* and *Verrucomicrobia,* whereas slightly lower levels in *Thaumarchaeota, Acidobacteria,* and *Actinobacteria-p* (Figure 6B and Supplementary Figure 4). For fungal phyla, *Mucoromycota* showed a significantly higher abundance in all plots of the sub-trial without mineral N-supply (OWPs coupled with DIG) *versus* the same OWP combined with mineral fertilizer (sub-trial with mineral N-supply), except in the FYM-DIG treated plots (Figures 7A,C,D and Supplementary Figure 5). In contrast, *Ascomycota* only exhibited a significantly higher level in FYM-DIG (Figure 7B and Supplementary Figure 5). *Chytridiomycota* displayed higher abundance in BIO-DIG and FYM-DIG treated plots, whereas lower abundance in *Basidiomycota* compared with their respective counterpart treatments (BIO-MIN and FYM-MIN) (Figures 7A,B and Supplementary Figure 5).

## 4 Discussion

The combined use of organic amendments and mineral fertilizers has been described as an improving approach for crop yields more than the single use of either of these products, also exhibiting changes in soil microbial communities (Allam et al., 2022; Cui et al., 2018; Francioli et al., 2016; Yang et al., 2023). Moreover, in the context of an agroecological transition aiming to reduce chemical fertilizer inputs, digestates could represent sustainable substitutes for mineral fertilizers. However, to our knowledge, no scientific studies have evaluated the effects of combining different OWP inputs with digestate applications on soil microbiota. The overall objective of this study was to assess the long-term cumulative effect of digestate application in field conditions on soil microbial communities (Archaea, Bacteria, and Fungi) by comparing different fertilization strategies based on two additional nitrogen supply approaches: (i) combining OWPs with raw digestate and (ii) combining the same OWPs with mineral fertilizer (i.e., digestate and mineral fertilizer inputs act as additional nitrogen sources).

### 4.1 Soil chemical properties influenced by the fertilization strategy

In the present study, four different OWPs were applied, each exhibiting diverse physicochemical characteristics, primarily differing in organic carbon content, index of residual carbon (Iroc), C/N ratio, and pH values. Indeed, the applied OWPs can be categorized into two groups: (i) OWPs with amending characteristics (BIO and FYM), which are rich in carbon content with high stable and recalcitrant organic matter (Iroc) and average C/N ratios between 12 and 18, and (ii) OWPs with fertilizing characteristics (SLU and DIG), which are liquid products with lower organic carbon content and C/N ratios ranging from 3 to 7.

Regarding the combination of organic and mineral fertilizers, previous studies performed in field experiments following years of fertilizer inputs have shown that combining organic products (e.g., manure, compost) and inorganic inputs increases SOC content compared to purely inorganic fertilization (Cui et al., 2018; Francioli et al., 2016; Kurzemann et al., 2020; Yue et al., 2016). Furthermore, multi-year field trials have reported no increases in SOC following repeated applications of sole digestate inputs (Bhogal et al., 2018; Odlare et al., 2008; Pastorelli et al., 2021; Šimon et al., 2015). In line with these studies, our results demonstrated that the repeated application of diverse OWPs (BIO, FYM, and SLU) combined with digestate (DIG) (plots BIO-DIG, FYM-DIG, and SLU-SIG) resulted in higher SOC contents compared with their respective counterpart treatment receiving additional N-supply via mineral fertilizer (plots BIO-MIN, FYM-MIN, and SLU-MIN), and control plots treated only with mineral fertilizer (MIN) exhibited the lowest SOC content. However, the maintenance of soil organic C stocks is mainly observed in plots receiving amending products combined with digestate (BIO-DIG and FYM-DIG treated plots), which is primarily attributed to the large quantity of organic carbon applied (Supplementary Figure 2) and the combination of products with stable and recalcitrant organic matter content (I_ROC_), confirming that in the long-term the C proportion contributing to SOC storage differed among OWPs. Indeed, I_ROC_ has been previously highlighted as a valuable indicator to estimate the contribution of OWPs to soil C stocks (Chen et al., 2022). Our findings align with previous research, demonstrating that organic nitrogen sources, particularly digestate, can effectively substitute synthetic fertilizers while significantly influencing soil nitrogen dynamics (Chen et al., 2022). As further detailed by Chen et al. (2022), the differences in N fertilizer replacement values (NFRV) among OWPs underscore the role of OWP type in modulating nutrient availability and ensuring long-term soil fertility. Moreover, regarding changes in soil pH, although a slight trend of soil acidification was observed in plots from the sub-trial with mineral nitrogen supply, a significant effect between counterpart treatments was observed only in the SLU-treated plots. Indeed, the application of an OWP with a low C/N ratio as SLU, combined with a mineral fertilizer could enhance soil acidification due to the rapid release of ammonium and subsequent nitrification, which releases hydrogen ions contributing to a decrease in soil pH (Dai et al., 2021). Overall, our results revealed that the fertilization strategy lastingly modified soil physicochemical properties, highlighting discrimination by sub-trial (strategy of additional N-supply source) and treatment (Supplementary Figure 3), which could become strongly differentiated in the coming years because changes are gradual and slow, intimately linked with biological soil quality and input materials quality (Krause et al., 2022).

### 4.2 Soil microbial shifts in response to the fertilization strategy

In most long-term field studies assessing the effects of combined organic and inorganic fertilizers on soil microbial communities, farmyard manure supplemented with a mineral fertilizer is the most commonly used treatment (Francioli et al., 2016; Kurzemann et al., 2020; Sadet-Bourgeteau et al., 2022; Yue et al., 2016; Zhao et al., 2014). These studies generally highlighted that the combination of organic and mineral fertilizers increases soil microbial biomass compared to purely mineral or organic fertilization (Francioli et al., 2016; Kurzemann et al., 2020; Lori et al., 2017; Sadet-Bourgeteau et al., 2022; Yue et al., 2016; Zhao et al., 2014). Furthermore, regarding the effect of sole digestate inputs, various studies in field experiments have reported no significant increases in microbial biomass after years of repeated liquid or raw digestate application (Bhogal et al., 2018; Johansen et al., 2015; Pastorelli et al., 2021; Šimon et al., 2015). Our findings exhibited that the long-term cumulative effect of different fertilization strategies based on combining OWPs with an additional N-supply via an organic source (digestate) or a chemical source (mineral fertilizer) induced a similar effect on the soil molecular microbial biomass.

Regarding the alpha diversity indices of soil microbial communities under different fertilization regimes, several field studies have reported that prokaryotic diversity indices are significantly lower in soils fertilized with mineral fertilizers than those receiving organic amendments or a combination of organic–inorganic inputs. In contrast, no significant changes in fungal diversity indices have been observed (Bebber and Richards, 2022; Cui et al., 2023; Cui et al., 2018; Francioli et al., 2016; Shu et al., 2022). Similarly, studies in field experiments assessing the effects of repeated digestate application on soil bacterial and fungal alpha diversity indices have reported no significant changes compared to mineral fertilization (Coelho et al., 2020; Pastorelli et al., 2021; Tang et al., 2021). Our results highlighted some differences in microbial alpha diversity indices depending on the type of OWP applied (Cui et al., 2023; Sadet-Bourgeteau et al., 2022; Shu et al., 2022). However, we found no significant differences between plots receiving sole digestate inputs (DIG) or only mineral fertilizer inputs (MIN). Thus, amending products with higher C/N ratios (i.e., farmyard manure and compost) may enhance microbial diversity by providing abundant carbon sources (Cui et al., 2023). Consequently, our results might be explained by the agronomic objective of reaching equivalent yields in both sub-trials; thus, the restitution of crop residues (i.e., belowground and aboveground crop residues, cover crops) brings significant additional organic C inputs on all treatments, which influence the SOC dynamic, a crucial determinant of soil microbial biomass and diversity (Bastida et al., 2021; Hu et al., 2014). Indeed, the dynamics of C and crop yields at the PROspective field experiment have been recently reported, showing that the C contribution by crop residues represents more than 70% of total C inputs (Chen et al., 2022), which potentially buffering and masking the fertilization effect.

Previous studies in long-term field experiments have revealed changes in the structure of soil microbial communities, significantly differentiated between fertilization strategies (e.g., mineral, organic, combined organic-mineral) (Francioli et al., 2016; Sadet-Bourgeteau et al., 2018; van der Bom et al., 2018; Yang et al., 2023). In line with these studies, our findings demonstrated that the quality of the applied products induced lasting modifications in microbial community structure, thus also reflecting discrimination by sub-trial (strategy of additional N-supply source), indicating that, in the long-term, repeated application of an OWP combined with an additional N-supply via an organic source (digestate) or a chemical source (mineral fertilizer) modified differently the structure of both prokaryotic and fungal communities. However, these modifications varied significantly depending on the OWP applied, providing insights to distinguish the amending versus fertilizing effects of the OWPs and their combinations with organic or mineral additional inputs. The additional nitrogen source *via* DIG or MIN within couples of counterpart treatments exhibited a significant effect on the structure of microbial communities only in soils treated with biowaste compost (BIO) and farmyard manure (FYM), reflecting an enhanced effect on soil microbial communities when an amending OWP (BIO or FYM) is combined with an organic fertilizer product (digestate), which has been previously explained as higher N-biological stability carries by digestate inputs (Zilio et al., 2023).

Regarding the effects of mineral fertilizers and organic amendments on soil microbial composition in agroecosystems, microorganisms present in the organic materials added to soil are rapidly outcompeted by soil-derived microorganisms and therefore only marginally influence changes in community structure (Saison et al., 2006). Thus, the effect of inputs would be mainly due to the physicochemical characteristics of amendment rather that to amendment-borne microorganisms (Saison et al., 2006). Concerning combined organic–inorganic fertilization, it has been reported that microbial networks exhibit higher stability compared to those relying solely on inorganic or organic fertilization (Yang et al., 2023). Moreover, several studies have stated that organic amendments stimulate copiotrophic bacterial phyla such as *Proteobacteria* and *Firmicutes*, as well as the fungal phylum *Mucoromycota* [previously classified under the phylum *Zygomycota* (Spatafora et al., 2016)]. These groups prefer nutrient-rich environments, respond rapidly to increased resource availability, and are capable of degrading complex organic compounds (Cui et al., 2023; Francioli et al., 2016; Hartmann et al., 2015; Hartmann and Six, 2023). In contrast, soils that have not received organic amendments (unfertilized and minerally fertilized soils) exhibit distinct microbial communities, often characterized by slow-growing oligotrophic microorganisms such as *Acidobacteria*, which are commonly found in resource-limited environments (Cui et al., 2023; Francioli et al., 2016; Hartmann et al., 2015; Hartmann and Six, 2023). In line with these statements, although our results did not show drastic changes in microbial community composition at the phylum level, the N-source supply strategy demonstrated a slight increase in the differential abundance of the prokaryotic phylum *Firmicutes* and the fungal phylum *Mucoromycota* in soils receiving only digestate or OWPs combined with DIG, compared to plots that received purely mineral fertilizer or OWPs combined with MIN. However, it must be considered that even though the fertilization strategy (i.e., organic, mineral, or combined) influences soil microbial community composition, their cumulative effect could be buffered by other agricultural practices (e.g., restitution of crop residues) (Krause et al., 2022; Sadet-Bourgeteau et al., 2022).

## 5 Conclusion

This study presented the first comparative analysis in long-term field conditions of fertilization strategies that combine different organic waste products (OWPs)—biowaste compost, farmyard manure, and urban sewage sludge—with raw digestate, compared to combining the same OWPs with mineral fertilizer. Both digestate and mineral fertilizer serve as supplementary nitrogen sources. This study provides new insights into the cumulative effects of substituting mineral fertilizers with digestates on soil microbial communities and soil physicochemical parameters. Specifically, our results highlighted that combining organic amending products such as biowaste compost and farmyard manure (i.e., products rich in carbon content, with high stable and recalcitrant organic matter and C/N ratios above 10) with a raw digestate (an organic fertilizing product) is an improved fertilization practice. This approach maintains SOC contents, increases soil phosphorus and potassium content, and stimulates the soil microbial communities differently compared to additional nitrogen supplied via mineral fertilizer. The sustainable development of agroecosystems significantly depends on a better understanding of the complex responses of soil microbial communities to different fertilization regimes. Future research should continue to assess the long-term impact of digestate application on soil microbiota in real agronomic field conditions, considering associated agricultural practices.

## Data Availability

The datasets presented in this study can be found in online repositories. The names of the repository/repositories and accession number(s) can be found at: https://www.ebi.ac.uk/metagenomics/, PRJEB79399.

## Footnotes

^1^ https://valor-pro.hub.inrae.fr/presentation-de-l-observatoire-soere-pro

^2^ https://www6.hautsdefrance.inrae.fr/las

^3^ https://www2.dijon.inrae.fr/plateforme_genosol/
