# Supplementary material for: Long-term effects of combining anaerobic digestate with other organic waste products on soil microbial communities
Source: Front Microbiol. 2025 Jan 7;15:1490034. doi: 10.3389/fmicb.2024.1490034 (PMC11752920; doi:10.3389/fmicb.2024.1490034)
Supplement: Supplementary file 1 [file Data_Sheet_1.PDF]

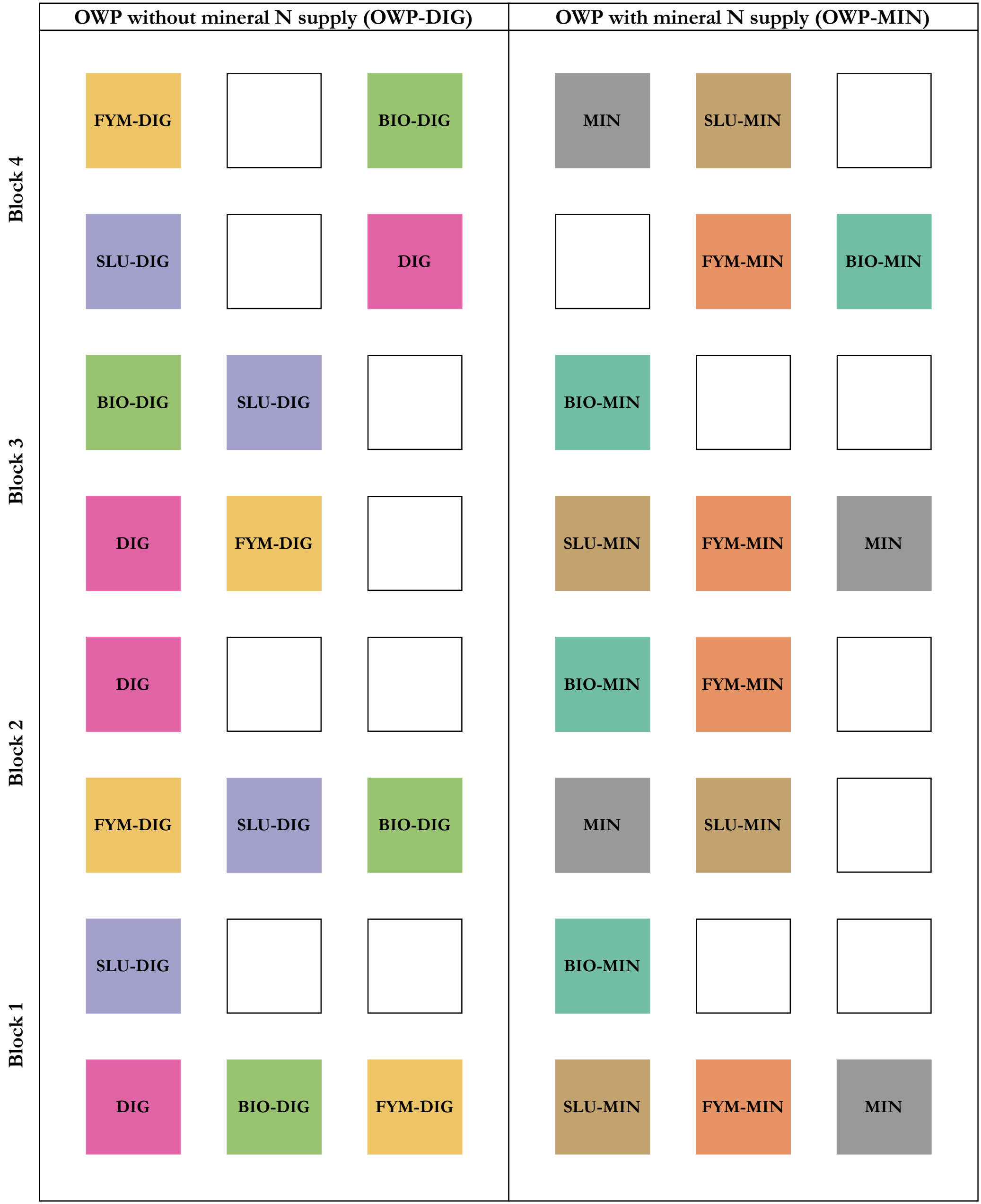
OWP without mineral N supply (OWP-DIG)
OWP with mineral N supply (OWP-MIN)
Block 4
Block 3
Block 2
Block 1
FYM-DIG
BIO-DIG
MIN
SLU-MIN
SLU-DIG
DIG
FYM-MIN
BIO-MIN
BIO-DIG
SLU-DIG
BIO-MIN
DIG
FYM-DIG
SLU-MIN
FYM-MIN
MIN
DIG
BIO-MIN
FYM-MIN
FYM-DIG
SLU-DIG
BIO-DIG
MIN
SLU-MIN
SLU-DIG
BIO-MIN
DIG
BIO-DIG
FYM-DIG
SLU-MIN
FYM-MIN
MIN
