# Supplementary figures and images for: Long-term effects of combining anaerobic digestate with other organic waste products on soil microbial communities
Source: Front Microbiol. 2025 Jan 7;15:1490034. doi: 10.3389/fmicb.2024.1490034 (PMC11752920; doi:10.3389/fmicb.2024.1490034)

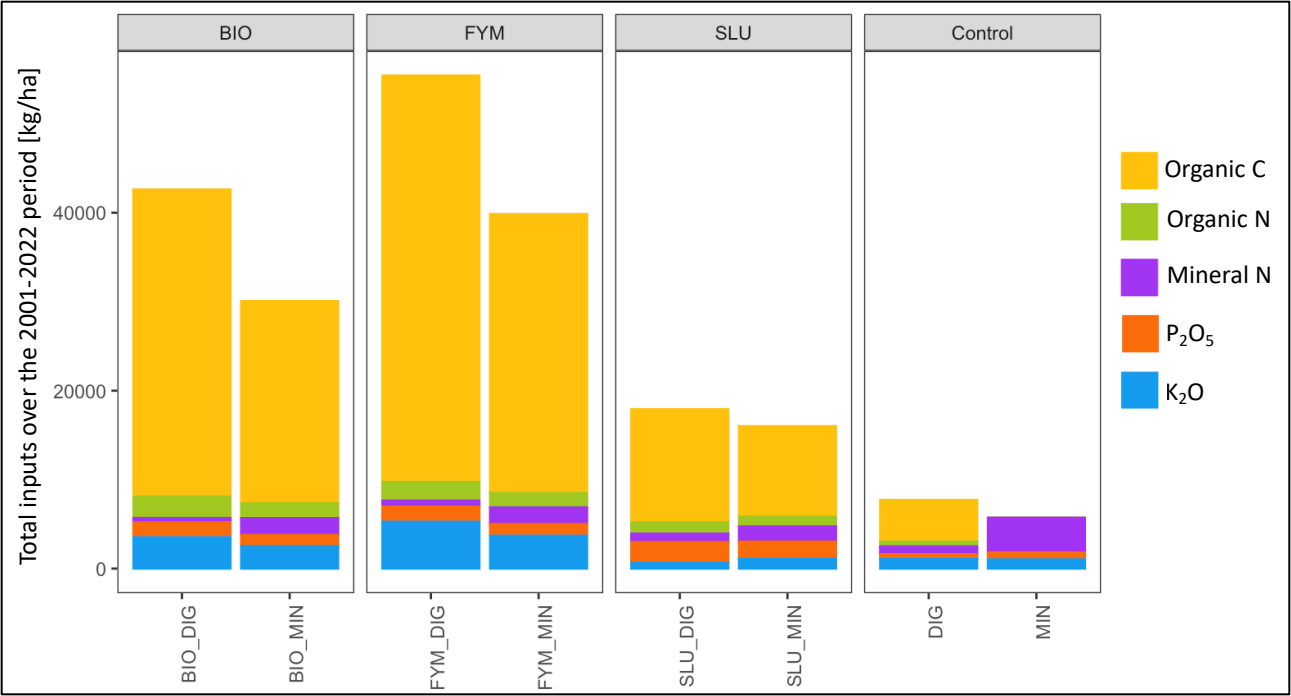

Supplement: Supplementary file 2 [file Data_Sheet_2.PDF]

Variables - PCA

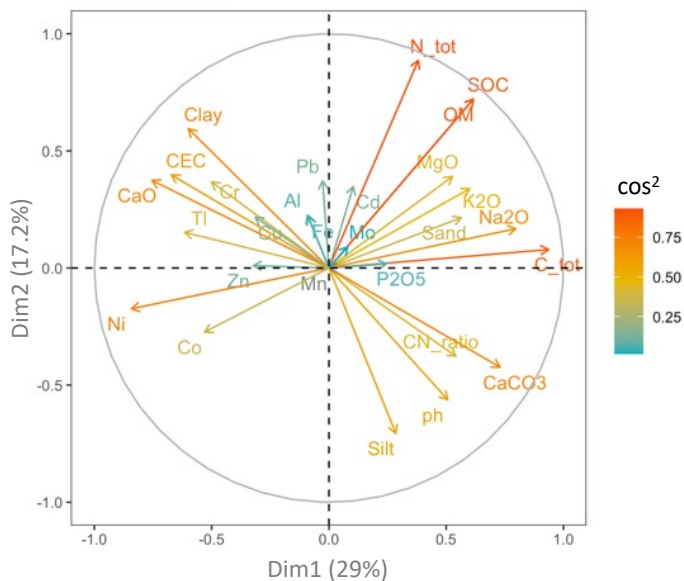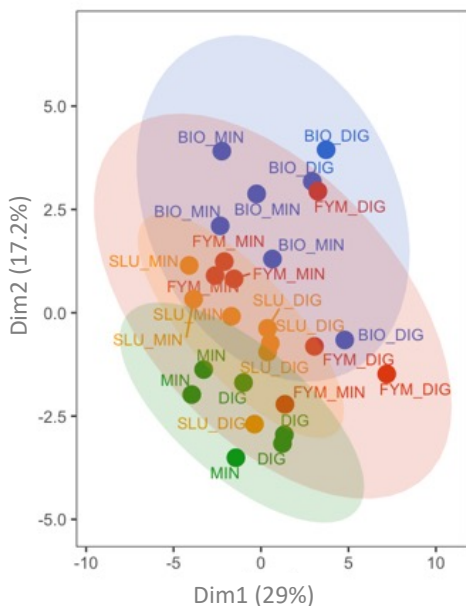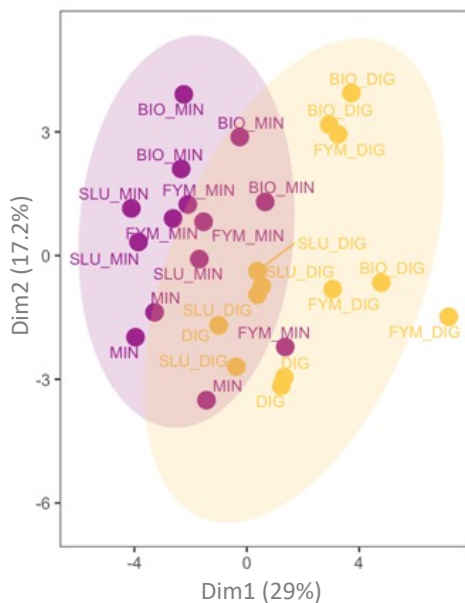

### Couples of counterpart treatments

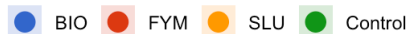

### Sub-trial

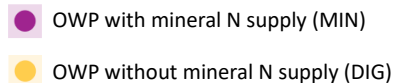

Supplement: Supplementary file 3 [file Data_Sheet_3.PDF]

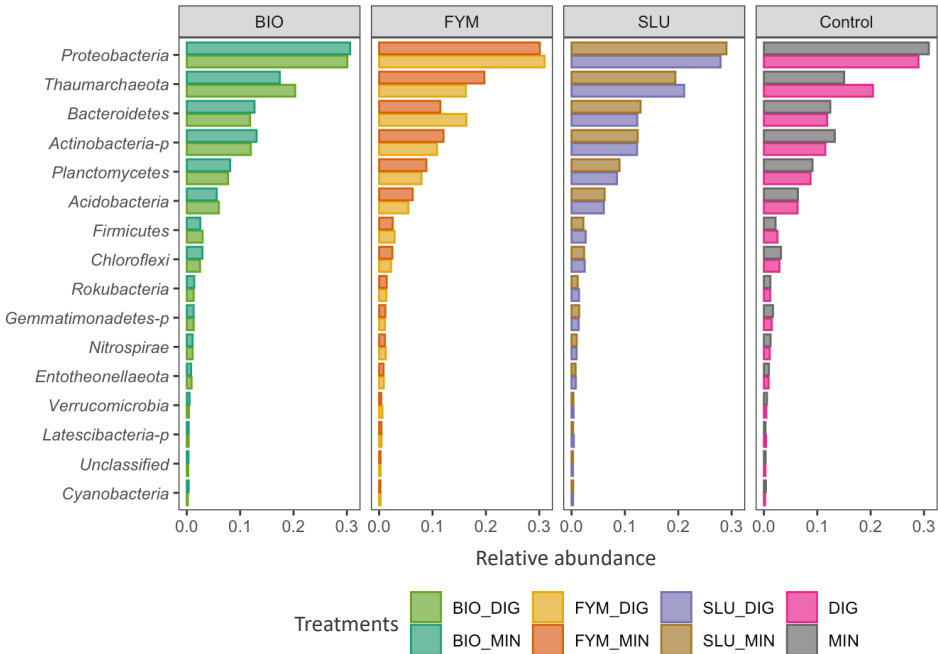

Supplement: Supplementary file 4 [file Data_Sheet_4.PDF]

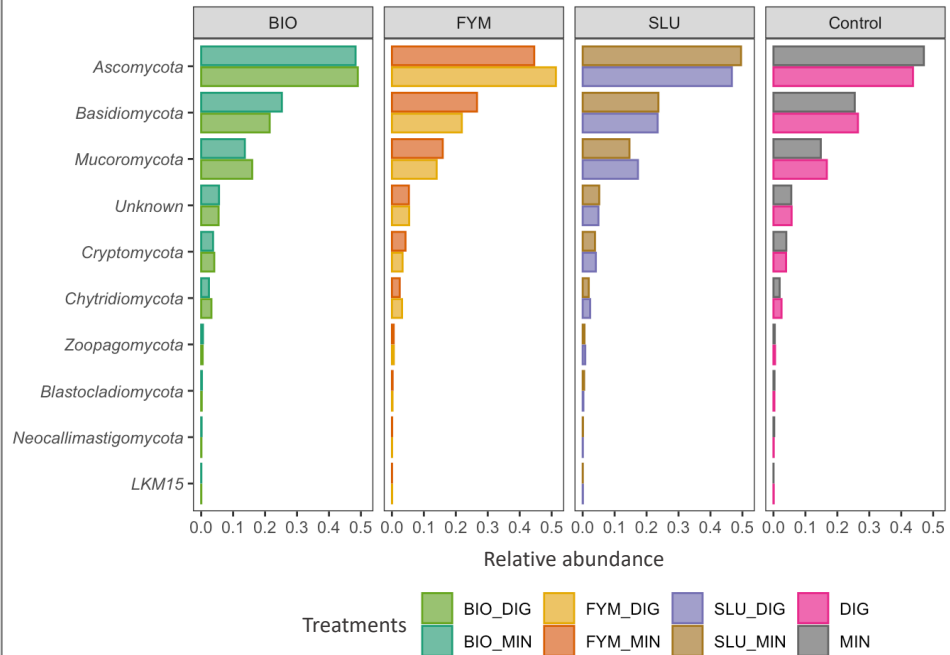

Supplement: Supplementary file 5 [file Data_Sheet_5.PDF]
